# Excess circulating angiopoietin-2 is a strong predictor of mortality in critically ill medical patients

**DOI:** 10.1186/cc7130

**Published:** 2008-11-21

**Authors:** Philipp Kümpers, Alexander Lukasz, Sascha David, Rüdiger Horn, Carsten Hafer, Robert Faulhaber-Walter, Danilo Fliser, Hermann Haller, Jan T Kielstein

**Affiliations:** 1 Department of Nephrology & Hypertension, Hannover Medical School, Carl-Neuberg-Strasse 1, Hannover, D-30171, Germany; 2 Department of Gastroenterology, Hepatology and Endocrinology, Hannover Medical School, Carl-Neuberg-Strasse 1, Hannover, D-30171, Germany; 3 Renal and Hypertensive Diseases, Saarland University Medical Centre, Kirrberger Straße, D-66421, Homburg/Saar, Germany

## Abstract

**Introduction:**

The endothelial specific angiopoietin (Ang)-Tie2 ligand-receptor system has been identified as a non-redundant mediator of endothelial activation in experimental sepsis. Binding of circulating Ang-1 to the Tie2 receptor protects the vasculature from inflammation and leakage, whereas binding of Ang-2 antagonises Tie2 signalling and disrupts endothelial barrier function. Here, we examine whether circulating Ang-1 and/or Ang-2 independently predict mortality in a cohort of critically ill medical patients.

**Methods:**

Circulating vascular endothelial growth factor (VEGF), Ang-1 and Ang-2 were prospectively measured in sera from 29 healthy controls and 43 medical ICU patients by immunoradiometric assay (IRMA) and ELISA, respectively. Survival after 30 days was the primary outcome studied.

**Results:**

Median serum Ang-2 concentrations were increasingly higher across the following groups: healthy controls, patients without sepsis, patients with sepsis and patients with septic shock. In contrast, Ang-1 and VEGF concentrations were significantly lower in all patient groups compared with healthy controls. Ang-2 correlated with partial pressure of oxygen in arterial blood (PaO_2_)/fraction of inspired oxygen (FiO_2_), tissue hypoxia, Sequential Organ Failure Assessment (SOFA) and Physiology and Chronic Health Evaluation II (APACHE II) score. Multivariate Cox regression analyses confirmed a strong independent prognostic impact of high Ang-2 as a novel marker of 30-day survival.

**Conclusions:**

A marked imbalance of the Ang-Tie system in favour of Ang-2 is present in critically ill medical patients. Our findings highlight the independent prognostic impact of circulating Ang-2 in critical illness. Ang-2 may be used as a readily available powerful predictor of outcome and may open new perspectives to individualise treatment in the ICU.

## Introduction

In critically ill patients, impaired vascular barrier function is a life-threatening feature that is causally determined by the activational state of the endothelial layer. In response to numerous different stimuli, 'quiescent' endothelial cells (anti-coagulant, anti-adhesive) undergo dramatic phenotypic changes towards an 'activated', pro-coagulant, pro-adhesive state, which is paralleled by disassembly of adherence junctions (e.g. VE-cadherin) and myosin driven cell contraction, resulting in inter-endothelial gap formation [1,2]. This highly regulated cascade of events results in net extravasation of fluid, a profound decrease in systemic vascular tone, collapse of the microcirculation and subsequent distributive shock, acute respiratory distress syndrome (ARDS) and eventually multiple organ dysfunction syndrome (MODS) [1,3-5]. Thus, an important goal in critical care medicine is to develop novel diagnostic and therapeutic strategies to address excess endothelial activation in the intensive care unit (ICU).

In 1996, Davis and colleagues discovered the angiopoietin (Ang)-Tie2 ligand-receptor system as the second class of vascular-specific receptor tyrosine kinases (the first being the vascular endothelial growth factor (VEGF)/VEGF-receptor system) [6]. Classically understood as an important regulator in vessel maturation and remodelling, recent studies demonstrated that the Ang-Tie2 system not only regulates angiogenesis, but also controls endothelial inflammation in a non-redundant manner [7-9].

Ang-1 and Ang-2 are antagonistic ligands that bind with similar affinity to the extracellular domain of the Tie2 receptor, which is almost exclusively expressed by endothelial cells. Binding of the agonist Ang-1 to the Tie2 receptor promotes vessel integrity, inhibits vascular leakage and suppresses inflammatory gene expression [10,11]. Constitutively expressed by pericytes and vascular smooth muscle cells, Ang-1 provides a stabilisation signal [8,12,13]. In contrast, Ang-2 inhibits binding of Ang-1 to Tie2, thereby disrupting protective Tie2 signalling [10,13-15]. Ang-2, which is considered the dynamic part of the Ang-Tie2 ligand-receptor, is stored and rapidly released by endothelial Weibel-Palade bodies [8]. Depending on the context, Ang-2 may act as a Tie2 agonist, especially in the presence of VEGF [16-18]. Intriguingly, VEGF itself was first identified and characterised as a potent stimulator of endothelial permeability and elevated circulating levels of VEGF seem to correlate with severity of sepsis and septic shock [19-21].

So far, several studies have investigated circulating Ang-1 and Ang-2 levels in critically ill patients [21-26]. Elevated Ang-2 concentrations correlate with the severity of illness as assessed by injury severity score [22], organ failure index [24], Acute Physiology and Chronic Health Evaluation (APACHE) II scores and Sequential Organ Failure Assessment (SOFA) scores [23,25,26]. In a recent study, we established and validated two novel immunoassays for the detection of circulating Ang-1 and Ang-2 in critically ill patients [27]. Despite the growing body of evidence indicating a role for Ang-2 as a mediator in critically illness, the value of Ang-2 as a predictive marker of outcome is poorly defined.

The aim of this study was to investigate the independent value of circulating Ang-1 and Ang-2 as predictors of outcome in critically ill medical patients.

## Materials and methods

### Patients

From the ICU at the Internal Medicine Department at Hannover Medical School, Germany, a tertiary care university hospital, 43 patients were enrolled at the time of ICU admission and studied prospectively. Patients were subdivided into the following groups: severe sepsis (n = 12), septic shock (n = 17) and critically ill patients (n = 14) with no evidence or suspicion of bacterial infection or sepsis (SCCM/ESICM/ACCP/ATS/SIS definitions [28]). Enrollment was performed in a consecutive fashion after obtaining written informed consents from the patients or their legal representatives. If the patient was recovering and able to communicate, he/she was informed of the study purpose and consent was required to further maintain status as study participant. The study was performed in accordance with the declaration of Helsinki and approved by the institutional review board. There were no co-morbidities that led to exclusion, except for age younger than 18 years or older than 75 years, being pregnant and having a malignant neoplasm.

Subjects were ventilated in accordance with the ARDSNet-derived protocol [29]. In 29 patients, invasive haemodynamic monitoring was performed by the Pulse contour Continous Cardiac Output (PiCCO) system (Pulsion Medical Systems, Munich, Germany) in addition to standard techniques. This device enables invasive on-line monitoring of several haemodynamic parameters, such as mean arterial pressure (MAP), heart rate (HR), cardiac index (CI), systemic vascular resistance index (SVRI), intrathoracic blood volume index (ITBVI) and extravascular lung water index (EVLWI), based on a transpulmonary thermodilution technique [30,31]. All relevant laboratory and medical data, including APACHE II [32] and SOFA scores [33], were obtained at the time of enrollment. Detailed patients' characteristics, including demographic, clinical and laboratory parameters, are shown in Table 1.

**Table 1:** Demographic, clinical and laboratory characteristics of patients

**Characteristics**	**Total**	**Non-septic patients**	**Severe sepsis**	**Septic shock**
**Number of patients**	43	14	12	17
Male	25 (59%)	6 (43%)	5 (42%)	14 (82%)
Female	18 (41%)	8 (57%)	7 (58%)	3 (18%)
**Age **(years, median (min – max)	51 (21 to 73)	59 (37 to 73)	51 (43 to 69)	51 (39 to 64)
**Reason for medical ICU admission**				
Pulmonary	15 (35%)	4 (29%)	3 (25%)	8 (47%)
Abdominal	10 (23%)	2 (14%)	4 (33%)	4 (24%)
Urogenital/retroperitoneal	3 (7%)	1(7%)	2 (17%)	0 (0%)
Cardiac	4 (9%)	3 (21%)	0 (0%)	1 (6%)
Cerebrovascular	4 (9%)	4 (29%)	0 (0%)	0 (0%)
Bloodstream infections	4 (9%)	0 (0%)	2 (17%)	2 (12%)
Miscellaneous	3 (7%)	0 (0%)	1 (8%)	2 (12%)
**Mean arterial pressure **(mmHg)	70 (40 to 96)	67 (53 to 84)	76 (67 to 91)	72 (60 to 81)
**Heart rate **(bpm)	100 (50 to 145)	102 (88 to 120)	90 (78 to 110)	106 (87 to 129)
**Noradrenaline **(μg/kg/min)	0.19 (0.0 to 1.96)	0.025 (0.0 to 0.07)	0.115 (0.02 to 0.18)	0.57 (0.32 to 0.77)
**Mechanically ventilated**, no.	36 (84%)	6 (43%)	12 (100%)	17 (100%)
FiO_2 _(%)	45 (26 to 100)	40 (34.53)	42 (35 to 62)	50 (59 to 60)
PaO_2_/FiO2	240 (68 to 640)	269 (218 to 367)	200 (130 to 257)	190 (138 to 272)
**CRP (mg/L)**	129 (51 to 268)	117 (5 to 194)	172 (79 to 304)	136 (54 to 282)
**Creatinine (mmol/L)**	251 (160 to 401)	116 (54 to 302)	354 (210 to 431)	273 (188 to 427)
**Lactate (mmol/L)**	1.9 (1.2 to 2.9)	1.3 (0.9 to 2.0)	1.6 (1.0 to 2.1)	2.9 (2.1 to 10.6)
**APACHE II score**	30 (6 to 48)	26 (17 to 30)	32 (25 to 35)	32 (29 to 38)
**SOFA score**	16 (1 to 22)	8 (4 to 11)	17 (14 to 20)	18 (16 to 20)
**Mortality**	25 (59%)	4 (29%)	8 (67%)	13 (77%)

APACHE II = Acute Physiology And Chronic Health Evaluation score; CRP = C-reactive protein; FiO_2 _= fraction of inspired oxygen; ICU = intensive care unit; PaO_2 _= partial pressure of oxygen in arterial blood; SOFA = Sequential Organ Failure Assessment score.

### Controls

Twenty-nine age- and gender-matched healthy volunteers from the Hannover Medical School staff served as controls (16 males, 13 females; age 58 (25 to 73 years)).

### Sampling

Serum samples for quantification of Ang-1, Ang-2 and VEGF were obtained at the time of enrollment, immediately placed on ice, centrifuged and stored at -80°C. All measurements were performed in a blinded fashion by the same investigator.

### Quantification of circulating Ang-1 and Ang-2

Ang-1 and Ang-2 were measured by in-house Immuno Radiometric Sandwich Assay (IRMA) and ELISA, respectively as previously described [27,34]. Polyclonal, anti-human Ang-1 affinity purified goat immunoglobulin (Ig) G and a monoclonal anti-human Ang-1 mouse antibody were obtained from R&D Systems (R&D, Oxford, UK). Recombinant human Ang-1 was purchased from Sigma-Aldrich (Sigma-Aldrich, Munich, Germany). Recombinant human Ang-2 monoclonal Ang-2 antibody and anti-Ang-2 antibody were purchased from R&D Systems (R&D, Oxford, UK).

### Quantification of circulating VEGF

Serum VEGF was measured using a sandwich ELISA kit according to the manufacturer's instructions (R&D Systems, Minneapolis, USA). This assay measures biologically active VEGF_121 _and VEGF_165_.

### Statistical analysis

Differences between patients and healthy controls were evaluated using a non-parametric Kruskal-Wallis test. The Mann-Whitney rank sum test was used for comparison between individual groups. Correlations between variables were assessed by the Spearman rank correlation coefficient. Pearson's correlation coefficient and linear regression analysis was performed after logarithmic transformation of Ang-2 values (logAng-2). The primary outcome studied was 30-day survival and was calculated from the day of ICU admission to death. Patients who survived the follow-up period were censored at day 30. Parameters independently associated with survival were identified by univariate and multivariate Cox proportional hazards models.

Variables found to be statistically significant at a 10% level in the univariate analysis were included in the multivariate model using backward elimination. Different models were established, incorporating either Ang-2, logAng-2 or the Ang-2/Ang-1 ratio, respectively. Two-sided p-values < 0.05 were considered statistically significant for all statistical procedures used. The distribution of the time-to-event variables were estimated using the Kaplan-Meier method with log-rank testing. Receiver operator characteristics (ROC) procedures were used to identify optimal cut-off values. Data are displayed as median and range (minimum to maximum) unless otherwise stated. All statistical analyses were performed with the SPSS package (SPSS Inc., Chicago, IL, USA) and the GraphPad Prism software (GraphPad Prism Software Inc. San Diego, California, USA).

## Results

### Decreased Ang-1 and VEGF concentrations and increased Ang-2 concentrations in critically ill medical patients

**Figure 1:**
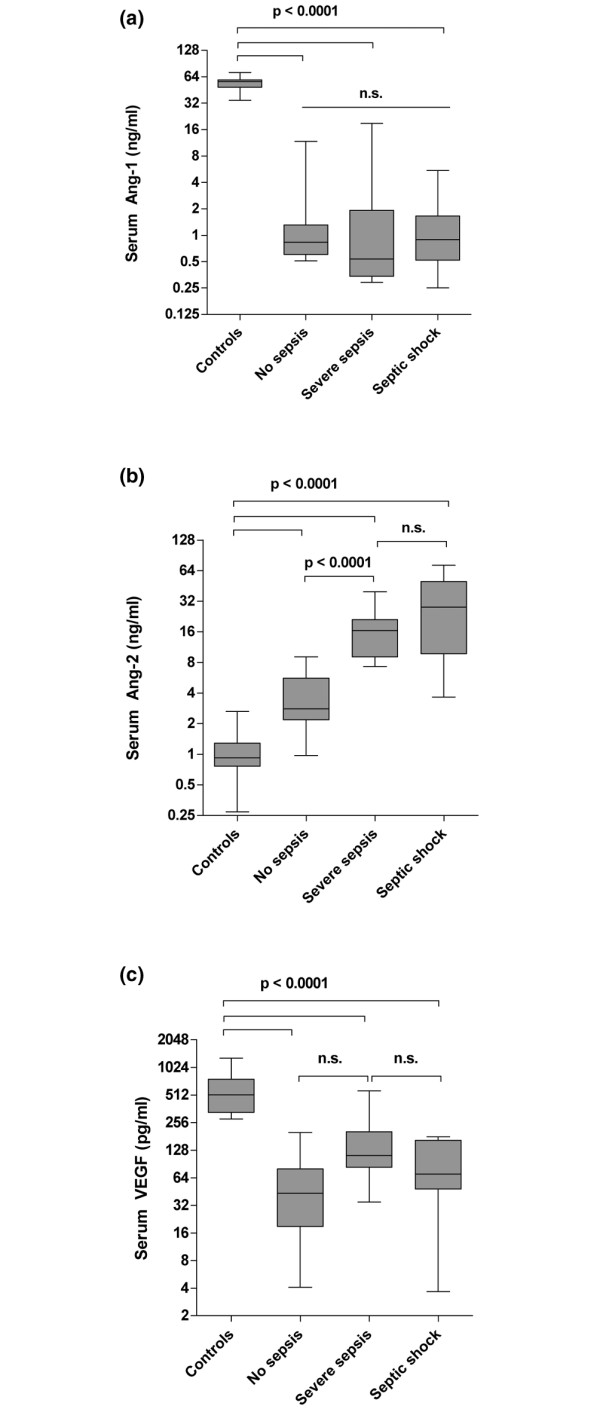
**Box plots of results in healthy controls and study patients**. Circulating (a) Angiopoietin (Ang) 1, (b) Ang-2 and (c) vascular endothelial growth factor (VEGF) serum concentrations in healthy controls (n = 29), critically ill patients without infection (no sepsis; n = 14), patients with severe sepsis (n = 12) and septic shock (n = 17). Horizontal bars indicate median values.

Ang-1 concentrations in critically ill non-septic patients (0.8 ng/ml, 0.5 to 11.7 ng/ml), patients with severe sepsis (0.5 ng/ml, 0.3 to 18.8 ng/ml) and patients with septic shock (0.9 ng/ml, 0.3 to 5.5 ng/ml were markedly decreased compared with healthy controls (56.4 ng/ml, 34.5 to 71.3 ng/ml, p < 0.0001) (Figure 1a). Ang-1 concentrations were no different between severe sepsis, septic shock and non-septic patients.

In contrast, median serum Ang-2 concentrations were consistently increased in critically ill non-septic patients (2.8 ng/ml, 1.0 to 9.0 ng/ml), in patients with severe sepsis (16.45 ng/ml, 2.7 to 39.7 ng/ml) and patients with septic shock (28.1 ng/ml, 3.7 to 72.6 ng/ml), compared with healthy controls (0.9 ng/ml, 0.3 to 2.6 ng/ml; all p < 0.0001 versus controls) (Figure 1b). Ang-2 was higher in patients with sepsis compared with non-septic patients (both p < 0.0001). Ang-2 concentrations were not different between patients with severe sepsis and septic shock (p = 0.12). Ang-1 and Ang-2 concentrations were neither linked to gender (Mann-Whitney test: p = 0.42 and p = 0.51) nor age (Spearman correlation: p = 0.83 and p = 0.24).

VEGF concentrations were markedly lower in critically ill non-septic patients (43.5 pg/ml, 4.1 to 200.0 pg/ml), patients with severe sepsis (112.7 pg/ml, 34.9 to 569.1 pg/ml) and patients with septic shock (70.5 pg/ml, 3.7 to 179.9 pg/ml compared with healthy controls (515.5 pg/ml, 280.6 to 1294.0 pg/ml, all p < 0.0001) (Figure 1c). VEGF concentrations were no different between patients with severe sepsis, patients with septic shock and non-septic controls. VEGF concentrations were not linked to gender (p = 0.67) and did not correlate with age (p = 0.33).

### Circulating Ang-2 concentrations correlate with SOFA and APACHE II scores

**Figure 2:**
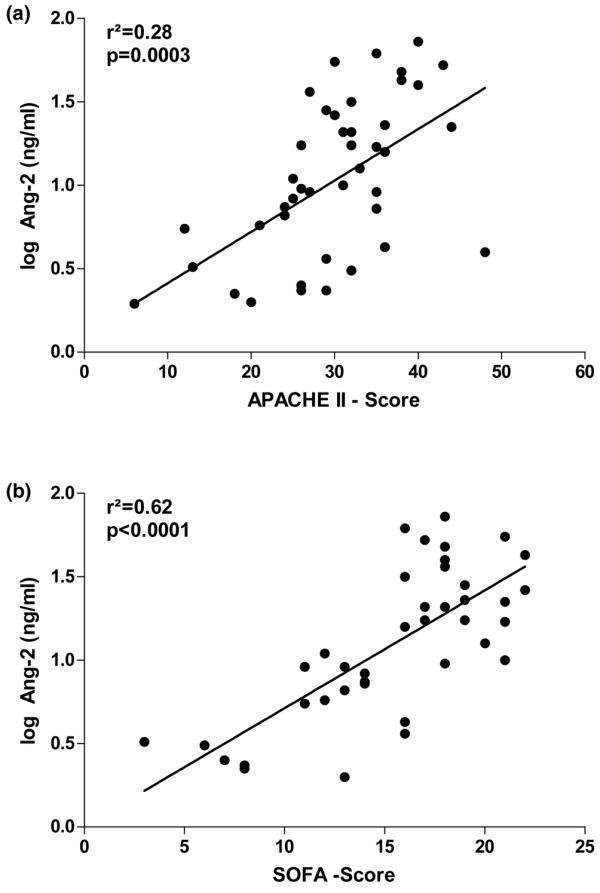
**Scatter plots showing correlations of results**. Correlations of Ang-2 serum concentrations with (a) the Acute Physiology and Chronic Health Evaluation (APACHE) II score and (b) the Sequential Organ Failure Assessment (SOFA) score in 43 critically ill patients (non-septic patients (n = 14); severe sepsis (n = 12) and septic shock (n = 17)).

Linear regression analysis detected a strong association of logAng-2 concentration with APACHE II scores (r^2 ^= 0.28, p = 0.0003) and SOFA scores (r^2 ^= 0.62, p < 0.0001) (Figures 2a,b; n = 43). Hypoxia has been shown to induce the release of Ang-2 from endothelial cells in preclinical models [35,36]. Of note, a strong correlation between Ang-2 concentrations and lactate levels as a surrogate marker for tissue hypoperfusion and microcirculatory tissue hypoxia was detected (r^2 ^= 0.25, p = 0.0007). Neither Ang-1 nor VEGF correlated with APACHE II scores, SOFA scores or C-reactive protein (CRP) levels.

### Association of Ang-1, Ang-2 and VEGF with pulmonary function and haemodynamics

Pre-clinical models have impressively demonstrated that the intact Ang-1/Tie2 signalling protects from ARDS in experimental sepsis [36-38]. We therefore examined the association between several parameters of haemodynamic and pulmonary function with circulating Ang-1, Ang-2 and VEGF levels. Of those, only Ang-2 showed an inverse correlation with partial pressure of oxygen in arterial blood (PaO_2_)/fraction of inspired oxygen (FiO_2_) (r^2 ^= -0.31; p = 0.046), and PaO_2 _(r^2 ^= -0.35; p = 0.023) as surrogate markers for ventilator support and pulmonary function. No association was seen for peak airway pressure (p = 0.6) or positive end expiratory pressure levels (p = 0.45). In addition to routine invasive haemodynamic monitoring (n = 43), 29 ventilated patients without atrial fibrillation qualified for detailed haemodynamic assessment by transpulmonary thermodilution technique (PiCCO system). Surprisingly, none of the measured angiogenic factors correlated with any of the haemodynamic parameters (MAP, CI, EVLWI, ITBVI, SVRI, vasopressor dose or central venous pressure; data not shown). The same results were obtained for invasive routine monitoring in all 43 patients (data not shown).

### Circulating Ang-2 predicts mortality in critically ill patients

To determine the relation between Ang-2 levels at admission and mortality, we initially performed univariate Cox proportional hazards analyses. In our whole cohort of critically ill medical patients, age, gender or the presence of sepsis did not show prognostic significance for survival (Table 2). The same was true for MAP, HR, CVP, urine output, noradrenaline dose, FiO_2_, PaO_2_/FiO_2_, thrombocytes, bilirubin, CRP and VEGF (Table 2). Among the tested variables, lactate (p = 0.006), APACHE II score (p = 0.013), SOFA score (p = 0.038) and the amount of circulating Ang-2 (p = 0.001) displayed prognostic significance (Table 2).

**Table 2:** Univariate and multivariate Cox regression analysis

	**Univariate**			**Multivariate**		
		
**Variables**	**HR**	**95% CI**	**p value**	**HR**	**95% CI**	**P value**
Age (years)	1.528	0.694 to 3.364	0.288			
Gender (m/f)	1.007	0.983 to 1.031	0.577			
Sepsis (yes/no)	2.688	0.918 to 7.874	0.072	1.004	0.154 to 6.553	0.997
						
MAP (mmHg)	0.981	0.954 to 1.009	0.186			
Heart rate (bpm)	0.995	0.976 to 1.014	0.583			
Noradrenaline (μg/kg/min)	1.433	0.695 to 2.954	0.330			
						
FiO_2 _(%)	1.002	0.980 to 1.025	0.873			
PaO_2_/FiO_2_	1.001	0.997 to 1.005	0.737			
						
CRP (mg/L)	0.998	0.995 to 1.002	0.364			
Lactate (mmol/L)	1.105	1.029 to 1.185	0.006*	1.064	0.986 to 1.148	0.111
						
APACHE II score	1.060	1.012 to 1.110	0.013*	1.040	0.985 to 1.099	0.154
SOFA score	1.107	1.006 to 1.219	0.038*	1.073	0.971 to 1.185	0.167
						
VEGF (pg/ml)	1.000	0.997 to 1.004	0.962			
Ang-1 (ng/ml)	1.010	0.918 to 1.111	0.840			
Ang-2 (ng/ml)^a^	1.034	1.013 to 1.056	0.001*	1.033	1.012 to 1.055	0.002*
Ang-2 (log10)^a^	4.383	1.628 to 11.802	0.003*	4.284	1.627 to 11.281	0.003*
Ang-2/Ang-1 (log10)^a^	2.630	1.207 to 5.729	0.015*	2.384	1.061 to 5.360	0.036* ^b^

*p < 0.05.

^a ^tested separately from each other;

^b ^In this model APACHE II remained significant in the multivariate model (p = 0.039).

Ang = Angiopoietin; APACHE II = Acute Physiology and Chronic Health Evaluation II; CI = confidence interval; CRP = C-reactive protein; f = female; FiO_2 _= fraction of inspired oxygen; HR = heart rate; m = male; MAP = mean arterial pressure; PaO_2 _= partial pressure of oxygen in arterial blood; SOFA = Sequential Organ Failure Assessment; VEGF = vascular endothelial growth factor.

Subsequently, the following variables were found to be statistically significant at a 10% level in the univariate analysis and subjected to multivariate Cox regression analysis: lactate, APACHE II score, SOFA score and circulating Ang-2 (Table 2). Except for Ang-2 (p = 0.002), all other variables did not remain significant in the multivariate setting (lactate (p = 0.111), APACHE II score (p = 0.154), SOFA score (p = 0.167)). The same results were obtained when either logAng-2 (p = 0.003) or the Ang-2/Ang-1 ratio (p = 0.036) were tested instead of Ang-2 (Table 2). Thus, circulating Ang-2 was identified as a strong, independent prognostic factor for 30-day survival in our cohort of critically ill medical patients. Given the context-dependent synergistic effects of Ang-2 and VEGF, we analysed various ratios incorporating Ang-1, Ang-2 and VEGF (data not shown). Except for the Ang-2/Ang-1 ratio, none of these models reached statistical significance (Table 2).

Ang-2 yielded an area under the ROC curve (AUC) value of 0.79 (standard error of the mean (SEM) = 0.07; 95% confidence interval = 0.65 to 0.93; p = 0.001). For comparison, the APACHE II score yielded an AUC value of 0.75 (SEM = 0.08; 95% confidence interval = 0.59 to 0.91; p = 0.005). A median circulating Ang-2 of more than 11.08 ng/ml predicted death with a specificity of 74% (95% confidence interval = 57 to 86) and a sensitivity of 67% (95% confidence interval = 54 to 77). The odds ratio for 30-day mortality was 5.6 (95% confidence interval = 1.5 to 20.5), positive and negative predictive values were 76% (95% confidence interval = 61 to 88) and 64% (95% confidence interval = 49 to 75), respectively.

**Figure 3:**
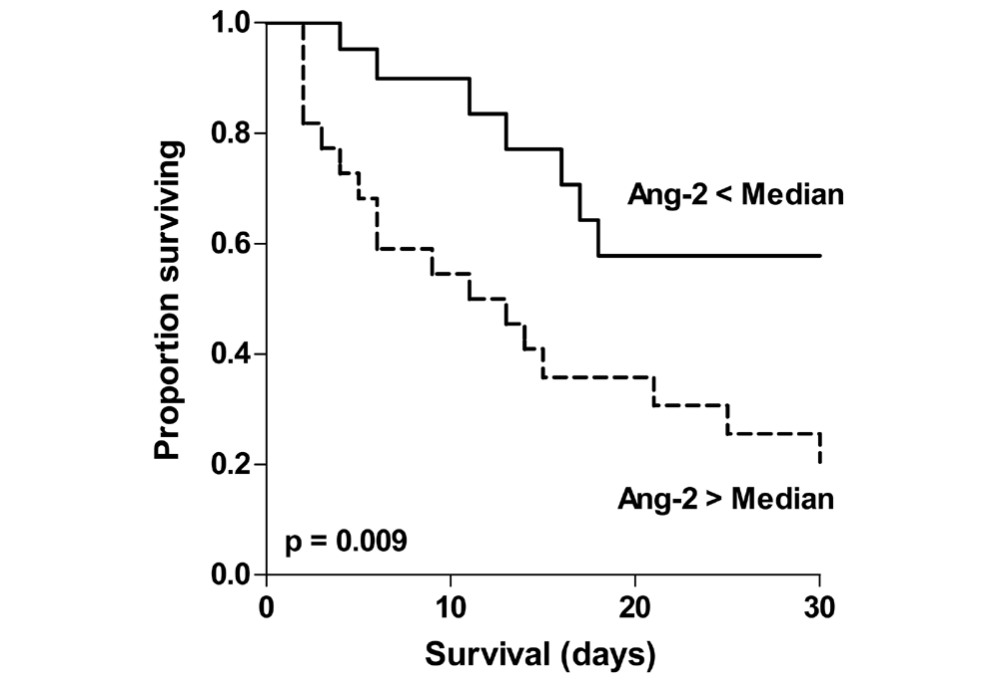
**Kaplan-Meier curves of survival stratified to Angiopoietin (Ang) 2**. (less versus greater than median; Log rank test p = 0.009).

Figure 3 illustrates the Kaplan-Meier curves of 30-day survival stratified to Ang-2 (less versus higher than median (11.08 ng/ml)). Log rank test confirmed statistical significance for Ang-2 (p = 0.009). Accordingly, the hazard for Ang-2 (> median) in our cohort was three-fold in the high Ang-2 (> 11.08 ng/ml) group compared with the low Ang-2 group (≤ 11.08 ng/ml). Of note, the 30-day survival of patients among the low Ang-2 group was 57%, while it was 20% in the group of patients with high Ang-2 levels.

## Discussion

The present study is a prospective clinical investigation of the prognostic value of circulating Ang-2 as a biomarker in critically ill patients. The results are that: critically ill patients are characterised by an excess of circulating Ang-2 in the presence of low Ang-1 and VEGF; Ang-2 correlates with severity of illness (APACHE II and SOFA scores), pulmonary dysfunction (PaO_2_/FiO_2 _and PaO_2_) and tissue hypoxia (lactate); using multivariate Cox proportional hazards regression analysis, Ang-2 was identified as the only independent predictor for survival in our cohort of medical ICU patients.

As a Weibel-Palade body-stored molecule, Ang-2 is released on endothelial stimulation by various factors, including complement, cytokines, fibrin, activated platelets and leucocytes, and changes in oxygenation or blood flow [8,15]. Orfanos and colleagues [25] and Ganter and colleagues [22] reported that in septic patients Ang-2 levels were associated with tumour necrosis factor-alpha levels and systemic hypoperfusion, respectively. In line with the latter finding, we detected a strong positive correlation between circulating Ang-2 and tissue hypoxia using lactate concentrations as a surrogate marker.

Little is known about the regulation of Ang-1 in critically ill patients. Experimental endotoxaemia has been shown to disrupt protective Ang-1/Tie-2 signalling by reducing Ang-1 and inducing Ang-2 expression [39]. In line with these results, admission levels of Ang-1 were markedly decreased in our patients. This finding is in apparent contrast to normal admission levels of Ang-1 in the aforementioned studies [22,23]. We assume that a decline in circulating Ang-1 is not an early feature in critically ill patients, but might reflect ongoing illness, as is often the case in medical patients compared with sudden onset of impairment in surgical and trauma patients. This interpretation fits with a recent cross-sectional study that showed low Ang-1 concentrations after lengthier mechanical ventilation in both, septic and non-septic ICU patients [21].

VEGF has been well characterised as an endothelial survival factor that prevents microvascular apoptotic cell loss *in vitro *[40]. In addition, the distinctive permeability-enhancing effects of VEGF underlie a significant role of this protein in acute vascular inflammation [41-43]. Both low and high VEGF concentrations have been found in critically ill patients, and its significance is not completely understood [21,44,45]. VEGF has been shown to modulate the effect of Ang-2 in a context-dependent fashion: when levels of VEGF are high, Ang-2 causes disassembly of inter-endothelial cell-cell contacts, whereas in the presence of low VEGF levels, Ang-2 induces endothelial cell death and vessel regression [16]. The latter constellation was present in our cohort, consistent with both a leaky and apoptotic endothelial cell phenotype in sepsis [1,46].

Consistent with previous reports, a strong positive correlation between Ang-2 concentrations and APACHE II and SOFA scores was detected in our cohort [23,25-27]. Thus it is reasonable to assume that individual Ang-2 levels may reflect the extent of activated endothelial surface among all organ-specific vascular beds at the same time.

In line with Parikh and colleagues [26] and van der Heijden and colleagues [21], we found a significant association of high Ang-2 concentrations with low PaO_2_/FiO_2 _and PaO_2 _values. This supports the idea that excess Ang-2 is involved in the increase in pulmonary permeability, leading to ARDS [26]. However, no correlation between EVLWI and Ang-2 or VEGF was present in our cohort. Likewise, no correlation between EVLWI and Ang-2 was detected by van der Heijden and colleagues [21]. EVLWI as a surrogate marker for endothelial is probably an imperfect tool to detect permeability in mechanically ventilated patients. Indeed, Ang-2 correlated with pulmonary leak index assessed by the ^67^Gallium-labelled tranferrin method [21]. Surprisingly, no such correlation could be detected for CI, MAP, CVP, surrogate parameters of pre-load (ITBVI) and after-load (SVRI), as well as for vasopressor support in the present study. These data reveal an important limitation for Ang-2 as a quantitative marker for vascular permeability: high Ang-2 might be a surrogate parameter for increased capillary permeability *per se*, but is a poor marker for the absolute extent of vascular 'leakiness'.

In contrast, we could identify Ang-2 as the strongest predictor for survival in our cohort of medical ICU patients using a multivariate Cox model. In a large trauma cohort study [22], Ang-2 correlated with mortality in a univariate analysis. In a surgical population with ARDS, Ang-2 predicted death with a similar discriminatory ability as the APACHE II score [23]. However, none of the aforementioned studies tested the independent predictive value of circulating Ang-2 compared with established predictors of outcome using a multivariate model. Ang-2 indeed outperformed the APACHE II and SOFA scores, as well as several other predictors in our cohort. If validated in larger cohorts, Ang-2 might be a promising new marker for early outcome prediction and decision-making in critically ill patients.

It should be pointed out that there are several limitations of our study. The sample size of the present study was small and the 95% confidence intervals for AUC are still wide. Also, we strictly included medical patients, thus our findings cannot be extrapolated to postoperative or surgical patient population. Future work will focus on the sensitivity and specificity between Ang-2 levels, severity scores, various cytokines and inflammatory markers in a larger ICU cohort including both, medical and surgical patients.

## Conclusion

In summary, a marked imbalance of the Ang/Tie system in favour of circulating Ang-2 is correlated with severity of illness and tissue hypoxia. High Ang-2 is probably a powerful independent biomarker of adverse clinical outcome in medical ICU patients. Further studies on the role of Ang-2 as a biomarker in critically ill patients are warranted.

## Key messages

• Ang-2 concentrations are increasingly higher across the following groups: healthy controls, patients without sepsis, sepsis and septic shock.

• Excess Ang-2 was independently associated with inferior survival.

• Ang-2 may be used as an early and readily available new biomarker in the ICU.

## Abbreviations

Ang: Angiopoietin; APACHE II: Acute Physiology and Chronic Health Evaluation II; ARDS: acute pulmonary distress syndrome; AUC: area under the curve; CI: cardiac index; CRP: C-reactive protein; ELISA: Enzyme Linked Immuno Sorbent Assay; EVLWI: extravascular lung water index; FiO_2_: fraction of inspired oxygen; HR: heart rate; ICU: intensive care unit; Ig: immunoglobulin; IRMA: immunoradiometric sandwich assay; ITBVI: intrathoracic blood volume index; MAP: mean arterial pressure; MODS: multiple organ dysfunction syndrome; PaO_2_: partial pressure of oxygen in arterial blood; PiCCO: Pulse contour Continous Cardiac Output; ROC: receiver operator characteristics; SEM: standard error of the mean; SOFA: Sequential Organ Failure Assessment; SVRI: systemic vascular resistance index; VEGF: vascular endothelial growth factor.

## Competing interests

The authors declare that they have no competing interests.

## Authors' contributions

PK had the initial idea, designed and supervised the research, analyzed the results, drew the diagrams and wrote the manuscript. AL established the immunoassays, performed the experiments, drew the diagrams and contributed to the manuscript. SD contributed to the idea, participated in the design of the study and contributed to the manuscript. RH established the immunoassays and supervised the experiments. CH, RF and DF identified patients, collected samples, provided clinical data and reviewed the manuscript. HH supervised the project and reviewed the manuscript. JTK designed and supervised the research, enrolled patients and reviewed the manuscript. PK and AL contributed equally to the work and are both considered first authors
